# Sharp bounds on sufficient-cause interactions under the assumption of no redundancy

**DOI:** 10.1186/s12874-017-0348-y

**Published:** 2017-04-21

**Authors:** Wen-Chung Lee

**Affiliations:** Research Center for Genes, Environment and Human Health and Institute of Epidemiology and Preventive Medicine, College of Public Health, National Taiwan University, Rm. 536, No. 17, Xuzhou Rd., Taipei, 100 Taiwan

**Keywords:** Sufficient component cause model, Epidemiologic methods, Causal inference, Interaction, Identifiability

## Abstract

**Background:**

Sufficient-cause interaction is a type of interaction that has received much attention recently. The sufficient component cause model on which the sufficient-cause interaction is based is however a non-identifiable model. Estimating the interaction parameters from the model is mathematically impossible.

**Methods:**

In this paper, I derive bounding formulae for sufficient-cause interactions under the assumption of no redundancy.

**Results:**

Two real data sets are used to demonstrate the method (R codes provided). The proposed bounds are sharp and sharper than previous bounds.

**Conclusions:**

Sufficient-cause interactions can be quantified by setting bounds on them.

**Electronic supplementary material:**

The online version of this article (doi:10.1186/s12874-017-0348-y) contains supplementary material, which is available to authorized users.

## Background

A common aim of many observational studies is to identify risk factors for disease. Once risk factors have been identified, researchers will often be interested in knowing whether any two factors can interact in causing the disease. ‘Sufficient-cause interaction’ (also referred to as ‘synergism’, ‘causal co-action’, ‘causal mechanistic interaction’, or simply ‘mechanistic interaction’) is a type of interaction that has received much attention recently [1–11] and is based on Rothman’s sufficient component cause model [12, 13]. The model posits that the causation of disease can be through any one of many different mechanisms or pathways. A mechanism/pathway requires several different component causes to operate, hence it is also called a ‘causal pie’. If two factors participate in the same causal pie, then a sufficient-cause interaction can be said to exist between them.

If the monotonicity assumption is not imposed [14–18], the sufficient component cause model in its general form is over-parameterized and non-identifiable. That is, the total number of model parameters exceeds the total degrees of freedom the data can offer. For example, two binary risk factors mean the data can offer at most four degrees of freedom (four different exposure profiles) but the model has a total of nine parameters, each corresponding to one of the nine possible causal-pie classes (one ‘all-unknown’ class unrelated to either factor, two main-effect classes for each factor, and four two-factor interaction classes). [If the monotonicity assumption is imposed on the two factors, the number of causal-pie classes reduces to four (one ‘all-unknown’ class unrelated to either factor, one main-effect class for each factor, and one two-factor interaction class), and the model becomes identifiable.] Researchers recently found ways to circumvent the non-identifiability problem and have developed methods *to test* for sufficient-cause interactions without imposing the monotonicity assumption [1–11]. It is however mathematically impossible *to estimate* the interaction parameters from a truly non-identifiable sufficient component cause model. At best, bounds can be set.

In this paper, I derive the bounding formulae for sufficient-cause interactions under the assumption of no redundancy [6–11, 19]. R codes for all computations are provided for convenience and the method is demonstrated with two real datasets. The proposed bounds will also be shown to be sharp and sharper than previous bounds [20].

## Methods

### Notations and definitions

This paper closely follows the notations used in previous studies [6–11]. Here, we are interested in the relationship between two exposures and a binary outcome (e.g., disease/no disease). We assume a population is studied from time 0 to *T*. The two exposures (*X*_1_ and *X*_2_) can have arbitrarily many levels (a total of *L*_1_ ≥ 2 and *L*_2_ ≥ 2, respectively). We assume that the exposure profile for a person does not change over time during the study period and is represented by profile = *x*_1_,*x*_2_, with *x*_1_ ∈ {1,…,*L*_1_} and *x*_2_ ∈ {1,…,*L*_2_} We assume that there is no loss to follow up and competing death during this study period. Let *D* = 1 represent disease occurrence in (0, *T*), and *D* = 0, otherwise. We assume *D* is known but the exact time of disease occurrence, if ever, is unknown to researchers. (*D* is a binary outcome within a defined period, not a time-to-event outcome.) It is assumed that there is no confounding, selection bias or measurement error in the study. The associations between the two exposures and the disease should reflect the genuine causal effects of the exposures on the disease.

While there is only a total of *L*_1_ × *L*_2_ exposure profiles, there is a total of (*L*_1_ + 1) × (*L*_2_ + 1) different causal-pie classes, including one all-unknown class, *L*_1_ + *L*_2_ main-effect classes, and *L*_1_ × *L*_2_ interaction classes. (Figure 1 in Lee’s paper [7] depicts (2 + 1) × (2 + 1) = 9 causal-pie classes in total for two binary exposures.) The causal-pie classes can be represented by class = *c*_1_,*c*_2_, with *c*_1_ ∈ {*,1,…,*L*_1_} and *c*_2_ ∈ {*,1,…,*L*_2_}. Note that here we introduce a null notation *, such that a class contains for *k* = 1,2, “*X*_*k*_ = *c*_*k*_” as one of its component causes if *c*_*k*_ ≠ *, and does not involve *X*_*k*_ whatsoever if *c*_*k*_ = *. For example, the all-unknown class involving neither *X*_1_ nor *X*_2_ is represented by class = *,*; the main-effect classes are represented by class = *c*_1_,* with *c*_1_ ≠ * for *X*_1_-only classes, and class = *,*c*_2_ with *c*_2_ ≠ * for *X*_2_-only classes; and the interaction classes are represented by class = *c*_1_,*c*_2_ with c_1_ ≠ * and c_2_ ≠ *.

The sufficient component cause model is partly deterministic and partly stochastic. The presence of risk factor(s) alone is not sufficient for the disease. Only when all unknown components (complement causes) also appear can the sufficient cause become complete and the disease occur. We let ${U}_{c_1,{c}_2}=1$ represent the arrival of the unknown components of the class = *c*_1_,*c*_2_ causal-pie class in (0, *T*), and ${U}_{c_1,{c}_2}=0$, otherwise, for *c*_1_ ∈ {∗, 1, …, *L*_1_} and *c*_2_ ∈ {∗, 1, …, *L*_2_}.

### Cumulative disease risk, cumulative completion risk, and relative prevalence

Let ${\mathrm{Risk}}^{\mathrm{profile}={x}_1,{x}_2}$ denote the cumulative disease risk in (0, *T*) for people in the population with profile = *x*_1_, *x*_2_, that is, Pr(*D* = 1|*X*_1_ = *x*_1_, *X*_2_ = *x*_2_). Let Risk_class = *i*,*j*_ denote the cumulative completion risk in (0, *T*) for a *specific* class = *i*, *j* sufficient-cause interaction, that is, Pr(*U*_*ij*_ = 1) for the specific *i* ∈ {1, …, *L*_1_} and *j* ∈ {1, …, *L*_2_}. Let Risk_class = int_ denote the cumulative completion risk in (0, *T*) for the *global* sufficient-cause interaction (sufficient-cause interaction regardless of classes), that is, $$ \Pr \left[{\displaystyle \underset{\begin{array}{l} i\in \left\{1,\dots, {L}_1\right\},\\ {} j\in \left\{1,\dots, {L}_2\right\}\end{array}}{\cup}\left({U}_{ij}=1\right)}\right] $$. Let Risk_class = any_ denote the cumulative completion risk over (0, *T*) for *any* class (all-unknown, main-effect, or interaction), that is, $$ \Pr \left[{\displaystyle \underset{\begin{array}{l} i\in \left\{\ast, 1,\dots, {L}_1\right\},\\ {} j\in \left\{\ast, 1,\dots, {L}_2\right\}\end{array}}{\cup}\left({U}_{ij}=1\right)}\right] $$, or equivalently, the proportion of those excluding the ‘immune’ persons in the study population during the study period. (An immune person is one who will *not* contract the disease during the study period, no matter what exposure profile he/she might contrary-to-fact assume.)

If the disease is rare we would always expect the above cumulative completion risks (or period prevalence, since these are defined for subjects in the study population over the study period) to be close to 0. To be informative for interactions for rare diseases, here we follow Sjölander et al.’s suggestion [20] to define the relative prevalence (RP) for the specific sufficient-cause interactions: ${\mathrm{RP}}_{\mathrm{class}= i, j}=\frac{{\mathrm{Risk}}_{\mathrm{class}= i, j}}{{\mathrm{Risk}}^{\mathrm{profile}= i, j}},$ for the specific *i* ∈ {1, …, *L*_1_} and *j* ∈ {1, …, *L*_2_}. In addition, we also define a relative prevalence for the global sufficient-cause interaction: ${\mathrm{RP}}_{\mathrm{class}=\mathrm{int}}=\frac{{\mathrm{Risk}}_{\mathrm{class}=\mathrm{int}}}{{\mathrm{Risk}}_{\mathrm{class}=\mathrm{any}}}.$ Note that specific and global RPs assume different denominators.

### The no-redundancy assumption

The no-redundancy assumption is a Poisson-like assumption which dictates there can only be at most one arrival event of the unknown components (at most one class of sufficient causes that can be completed) in a sufficiently short time interval for each and every subject in the population [19]. In other words, there are at most (*L*_1_ + 1) × (*L*_2_ + 1) + 1 causal response types in a very short time interval, with each of the (*L*_1_ + 1) × (*L*_2_ + 1) types corresponding to exactly one causal-pie class, plus an additional one for the immune type. The table in Lee’s paper [6] enumerates the total (2 + 1) × (2 + 1) + 1 = 10 causal response types for two binary exposures under the no-redundancy assumption. By comparison, the conventional potential outcome model (without the no-redundancy assumption) would have a total of ${2}^{L_1\times {L}_2}$ causal response types, and 2^2 × 2^ = 16 for two binary exposures.

The no-redundancy assumption is a relatively weak assumption that can still hold true even if there is a strong dependency in the arrival events. Note that no redundancy is specified only with respect to an infinitesimally short time interval. It says nothing about the entire follow-up period and can therefore also hold true even for non-rare diseases (diseases with high Risk^profile = *i*,*j*^ for *i* ∈ {1, …, *L*_1_} and *j* ∈ {1, …, *L*_2_}). Several sufficient-cause interaction tests had previously been developed under this assumption [6–11].

### Bounds on sufficient-cause interactions under the no-redundancy assumption

In Additional file 1, I derive the bounds on sufficient-cause interactions under the no-redundancy assumption. For the specific sufficient-cause interactions, the bounds are (LB in superscript for lower bound; UB for upper bound):

1 $$ {\mathrm{Risk}}_{\mathrm{class}= i, j}^{\mathrm{LB}}=1-\underset{\begin{array}{l}\left({i}^{\prime}\ne i\right)\in \left\{1,\dots, {L}_1\right\}\\ {}\left({j}^{\prime}\ne j\right)\in \left\{1,\dots, {L}_2\right\}\end{array}}{ \min}\left\{\frac{1-{\mathrm{Risk}}^{\mathrm{profile}= i, j}}{\left(1-{\mathrm{Risk}}^{\mathrm{profile}={i}^{\prime }, j}\right)\times \left(1-{\mathrm{Risk}}^{\mathrm{profile}= i,{j}^{\prime }}\right)},1\right\}, $$

2 $$ {\mathrm{Risk}}_{\mathrm{class}= i, j}^{\mathrm{UB}}={\mathrm{Risk}}^{\mathrm{profile}= i, j}, $$

3 $$ {\mathrm{RP}}_{\mathrm{class}= i, j}^{\mathrm{LB}}=\frac{{\mathrm{Risk}}_{\mathrm{class}= i, j}^{\mathrm{LB}}}{{\mathrm{Risk}}^{\mathrm{profile}= i, j}}, $$

and

4 $$ {\mathrm{RP}}_{\mathrm{class}= i, j}^{\mathrm{UB}}=1, $$

respectively, for the specific *i* ∈ {1, …, *L*_1_} and *j* ∈ {1, …, *L*_2_}. For the global sufficient-cause interaction, the bounds are:

5 $$ {\mathrm{Risk}}_{\mathrm{class}=\mathrm{int}}^{\mathrm{LB}}=1-\underset{\begin{array}{l}\mathrm{permutations}\ \mathrm{of}\ \left({u}_1,\dots, {u}_{L_1}\right)\\ {}\ \mathrm{and}\ \mathrm{permutations}\ \mathrm{of}\ \left({v}_1,\dots, {v}_{L_2}\right)\end{array}}{ \min}\left\{{\displaystyle \prod_{i=1}^{L_1}{\displaystyle \prod_{j=1}^{L_2}{\left(1-{\mathrm{Risk}}^{\mathrm{profile}= i, j}\right)}^{u_i\times {v}_j}}},1\right\}, $$

6 $$ {\mathrm{Risk}}_{\mathrm{class}=\mathrm{int}}^{\mathrm{UB}}=1-{\displaystyle \prod_{i=1}^{L_1}{\displaystyle \prod_{j=1}^{L_2}\left(1-{\mathrm{Risk}}^{\mathrm{profile}= i, j}\right)}}, $$

7 $$ {\mathrm{RP}}_{\mathrm{class}=\mathrm{int}}^{\mathrm{LB}}=\frac{{\mathrm{Risk}}_{\mathrm{class}=\mathrm{int}}^{\mathrm{LB}}}{1-{\displaystyle \prod_{i=1}^{L_1}{\displaystyle \prod_{j=1}^{L_2}\left(1-{\mathrm{Risk}}^{\mathrm{profile}= i, j}\right)}}}, $$

and

8 $$ {\mathrm{RP}}_{\mathrm{class}=\mathrm{int}}^{\mathrm{UB}}=1, $$

respectively. [Risk^LB^_class = int_ involves the use of ‘contrast coefficients’. The contrast coefficients for *X*_1_, $\left({u}_1,\dots, {u}_{L_1}\right)$, contains as its elements an equal number of ‘+1’ and ‘−1’ if *L*_1_ is an even number, and exactly one ‘0’ and an equal number of ‘+1’ and ‘−1’ for the remaining elements if otherwise. The contrast coefficients for *X*_2_, $\left({v}_1,\dots, {v}_{L_2}\right)$, are similarly constructed.]

When both exposures are binary, the lower bound formula is simplified considerably. Formula (1) becomes

9 $$ {\mathrm{Risk}}_{\mathrm{class}= i, j}^{\mathrm{LB}}=1- \min \left\{\frac{1-{\mathrm{Risk}}^{\mathrm{profile}= i, j}}{\left(1-{\mathrm{Risk}}^{\mathrm{profile}=3- i,\  j}\right)\times \left(1-{\mathrm{Risk}}^{\mathrm{profile}= i,3- j}\right)},1\right\}, $$

for *i*, *j* ∈ {1, 2}. Formula (5) becomes

10 $$ {\mathrm{Risk}}_{\mathrm{class}=\mathrm{int}}^{\mathrm{LB}}=1- \min \left(\mathrm{PRISM},\ {\mathrm{PRISM}}^{-1}\right), $$

where $\mathrm{PRISM}=\frac{\left(1-{\mathrm{Risk}}^{\mathrm{profile}=2,1}\right)\times \left(1-{\mathrm{Risk}}^{\mathrm{profile}=1,2}\right)}{\left(1-{\mathrm{Risk}}^{\mathrm{profile}=2,2}\right)\times \left(1-{\mathrm{Risk}}^{\mathrm{profile}=1,1}\right)}$ is the ‘peril ratio index of synergy based on multiplicativity’ [7].

### Case-control study for rare diseases

For a rare disease with exceedingly low risks, we have $1-\frac{1-{\mathrm{Risk}}^{\mathrm{profile}= i, j}}{\left(1-{\mathrm{Risk}}^{\mathrm{profile}= i\prime, j}\right)\times \left(1-{\mathrm{Risk}}^{\mathrm{profile}= i, j\prime}\right)}\approx {\mathrm{Risk}}^{\mathrm{profile}= i, j}-{\mathrm{Risk}}^{\mathrm{profile}= i\prime, j}-{\mathrm{Risk}}^{\mathrm{profile}= i, j\prime }$ for (*i*′ ≠ *i*) ∈ {1, …, *L*_1_} and (*j*′ ≠ *j*) ∈ {1, …, *L*_2_}, $1-{\displaystyle \prod_{i=1}^{L_1}{\displaystyle \prod_{j=1}^{L_2}{\left(1-{\mathrm{Risk}}^{\mathrm{profile}= i, j}\right)}^{u_i\times {v}_j}}}\approx {\displaystyle \sum_{i=1}^{L_1}{\displaystyle \sum_{j=1}^{L_2}{u}_i\times {v}_j\times {\mathrm{Risk}}^{\mathrm{profile}= i, j}}},$ and $1-{\displaystyle \prod_{i=1}^{L_1}{\displaystyle \prod_{j=1}^{L_2}\left(1-{\mathrm{Risk}}^{\mathrm{profile}= i, j}\right)}}\approx {\displaystyle \sum_{i=1}^{L_1}{\displaystyle \sum_{j=1}^{L_2}{\mathrm{Risk}}^{\mathrm{profile}= i, j}}}.$ Therefore, the lower bounds on the relative prevalence of sufficient-cause interactions are approximately

11 $$ \begin{array}{l}{\mathrm{RP}}_{\mathrm{class}= i, j}^{\mathrm{LB}}\approx \frac{{ \max}_{\begin{array}{l}\left({i}^{\prime}\ne i\right)\in \left\{1,\dots, {L}_1\right\}\\ {}\left({j}^{\prime}\ne j\right)\in \left\{1,\dots, {L}_2\right\}\end{array}}\left\{{\mathrm{Risk}}^{\mathrm{profile}= i, j}-{\mathrm{Risk}}^{\mathrm{profile}= i\prime, j}-{\mathrm{Risk}}^{\mathrm{profile}= i, j\prime },0\right\}}{{\mathrm{Risk}}^{\mathrm{profile}= i, j}}\hfill \\ {}\kern2.28em \approx \frac{{ \max}_{\begin{array}{l}\left({i}^{\prime}\ne i\right)\in \left\{1,\dots, {L}_1\right\}\\ {}\left({j}^{\prime}\ne j\right)\in \left\{1,\dots, {L}_2\right\}\end{array}}\left\{{\mathrm{OR}}^{\mathrm{profile}= i, j}-{\mathrm{OR}}^{\mathrm{profile}={i}^{\prime }, j}-{\mathrm{OR}}^{\mathrm{profile}= i,{j}^{\prime }},0\right\}}{{\mathrm{OR}}^{\mathrm{profile}= i, j}}\hfill \end{array} $$

for the specific *i* ∈ {1, …, *L*_1_} and *j* ∈ {1, …, *L*_2_}, and

12 $$ \begin{array}{r}\hfill {\mathrm{RP}}_{\mathrm{class}=\mathrm{int}}^{\mathrm{LB}}\approx \frac{\underset{\begin{array}{l}\mathrm{permutations}\ \mathrm{of}\ \left({u}_1,\dots, {u}_{L_1}\right)\\ {}\mathrm{and}\ \mathrm{permutations}\ \mathrm{of}\ \left({v}_1,\dots, {v}_{L_2}\right)\end{array}}{ \max}\left\{{\displaystyle \sum_{i=1}^{L_1}{\displaystyle \sum_{j=1}^{L_2}{u}_i\times {v}_j\times {\mathrm{Risk}}^{\mathrm{profile}= i, j}}},0\right\}}{{\displaystyle \sum_{i=1}^{L_1}{\displaystyle \sum_{j=1}^{L_2}{\mathrm{Risk}}^{\mathrm{profile}= i, j}}}}\\ {}\hfill \approx \frac{\underset{\begin{array}{l}\mathrm{permutations}\ \mathrm{of}\ \left({u}_1,\dots, {u}_{L_1}\right)\\ {}\mathrm{and}\ \mathrm{permutations}\ \mathrm{of}\ \left({v}_1,\dots, {v}_{L_2}\right)\end{array}}{ \max}\left\{{\displaystyle \sum_{i=1}^{L_1}{\displaystyle \sum_{j=1}^{L_2}{u}_i\times {v}_j\times {\mathrm{OR}}^{\mathrm{profile}= i, j}}},0\right\}}{{\displaystyle \sum_{i=1}^{L_1}{\displaystyle \sum_{j=1}^{L_2}{\mathrm{OR}}^{\mathrm{profile}= i, j}}}},\end{array} $$

where ${\mathrm{OR}}^{\mathrm{profile}= i, j}=\frac{{\mathrm{Odds}}^{\mathrm{profile}= i, j}}{{\mathrm{Odds}}^{\mathrm{profile}=1,1}}=\frac{{\mathrm{Risk}}^{\mathrm{profile}= i, j}}{1-{\mathrm{Risk}}^{\mathrm{profile}= i, j}}/\frac{{\mathrm{Risk}}^{\mathrm{profile}=1,1}}{1-{\mathrm{Risk}}^{\mathrm{profile}=1,1}}$ is the odds ratio comparing the profile = *i*, *j* subjects with the profile = 1, 1 subjects. These bounds are functions of odds ratios and can therefore be estimated directly from a case-control study conducted in the study population.

When both exposures are binary, the bounds reduce to

13 $$ {\mathrm{RP}}_{\mathrm{class}= i, j}^{\mathrm{LB}}\approx \frac{ \max \left\{{\mathrm{OR}}^{\mathrm{profile}= i, j}-{\mathrm{OR}}^{\mathrm{profile}=3- i,\  j}-{\mathrm{OR}}^{\mathrm{profile}= i,3- j},\ 0\right\}}{{\mathrm{OR}}^{\mathrm{profile}= i, j}}, $$

for the specific *i*, *j* ∈ {1, 2}, and

14 $$ {\mathrm{RP}}_{\mathrm{class}=\mathrm{int}}^{\mathrm{LB}}\approx \frac{\left|\mathrm{RERI}\right|}{{\mathrm{OR}}^{\mathrm{profile}=2,2}+{\mathrm{OR}}^{\mathrm{profile}=2,1}+{\mathrm{OR}}^{\mathrm{profile}=1,2}+1}, $$

where RERI = OR^profile = 2,2^ − OR^profile = 2,1^ − OR^profile = 1,2^ + 1 is the ‘relative excess risk due to interaction’ in terms of odds ratios [1–5].

Additional file 2 presents two functions written in R code: ‘bounds.cohort’ for cohort data and ‘bounds.cscn’ for case-control data. Input the data as the argument and the functions will output the various bounds on sufficient-cause interactions. Additionally, the functions also automatically perform 10,000 bootstrap replications to calculate a 95% lower confidence limit for a lower bound and a 95% upper confidence limit for an upper bound.

## Results

### Example 1. A cohort study of hypertension risk

The data of a cohort study on hypertension risk (taken directly from Example 3 in Zou’s paper [21]) is analyzed here as an example. The cohort study assesses the effects of body mass index (BMI, coded as 1 if BMI ≥ 25 kg/m^2^ and 0 if otherwise) and age (coded as 1 if age ≥ 40 years and 0 if otherwise) on hypertension (coded as 1 if diastolic blood pressure ≥ 90 mmHg and 0 if otherwise). We assume that there is no confounding, selection bias or measurement error in the study and that the follow-up is 100% complete.

Table 1 presents the bounds and their 95% bootstrapped confidence limits for sufficient-cause interactions between BMI and age. The lower bounds for the (high BMI, old age)-specific sufficient-cause interaction are greater than zero (0.0411 for the cumulative completion risk; 0.1509 for the relative prevalence), but do not achieve statistical significance (as judged from their 95% lower confidence limits which are both zero). As for the global sufficient-cause interactions, the lower bounds are 0.0830 (cumulative completion risk) and 0.1758 (relative prevalence), respectively, and are both significantly greater than zero. The upper bound for the cumulative completion risk of the global sufficient-cause interaction is 0.4718 with an upper 95% confidence limit of 0.4993.

**Table 1:** Bounds on sufficient-cause interactions in a cohort study on hypertension risk (Example 1)

	Case number	Population	Risk	Cumulative completion risk of sufficient-cause interaction				Relative prevalence of sufficient-cause interaction	
				95% LCL^c^	LB^c^	UB^c^	95% UCL^c^	95% LCL^c^	LB^c^
Specific (BMI^a^, age^b^)									
(low, young)	79	1810	0.0437	0.0000	0.0000	0.0436	0.0530	0.0000	0.0000
(low, old)	100	681	0.1468	0.0000	0.0000	0.1468	0.1733	0.0000	0.0000
(high, young)	153	1385	0.1105	0.0000	0.0000	0.1105	0.1278	0.0000	0.0000
(high, old)	278	1021	0.2723	0.0000	0.0411	0.2723	0.2997	0.0000	0.1509
Global	610	4897	0.1246	0.0338	0.0830	0.4718	0.4993	0.0879	0.1758

^a^old: age ≥ 40 years; young: age < 40

^b^ *BMI* body mass index; high: BMI ≥ 25 kg/m^2^; low: BMI < 25

^c^ *LCL* lower confidence limit for the lower bound, *LB* lower bound, *UB* upper bound, *UCL* upper confidence limit for the upper bound

### Example 2. A case-control study on lung cancer risk

Zhang et al.’s case-control data (directly taken from Table 4 in reference [22]) is analyzed here as the second example. The study examines the gene-gene interactions between two DNA base excision repair genes on lung cancer risk: the *ADPRT* (*adenosine diphosphate ribosyltransferas*e) Val762Ala polymorphism and the *XRCC1* (*X-ray repair cross-complementing group 1*) Arg366Gln polymorphism (both having three genotypes). The rare-disease assumption is invoked here (For lung cancer, the assumption is tenable). In addition, we assume gene-environment independence [10] such that unmeasured environmental factors, no matter what they may be, cannot confound the genetic effects of the two studied genes.

Table 2 presents the lower bounds and the 95% lower limits for sufficient-cause interactions between these two genes. The lower bound of the relative prevalence for the (*ADPRT* = *Ala/Ala*, *XRCC1* = *Gln/Gln*)-specific sufficient-cause interaction is greater than zero (0.5221) but does not achieve statistical significance. The lower bound of the relative prevalence for the global *ADPRT*-*XRCC1* interaction is 0.2471 and is significantly greater than zero (as judged from its 95% lower confidence limit which is 0.0784).

**Table 2:** Bounds on sufficient-cause interactions in a case-control study on lung cancer risk (Example 2)

	Case Number	Control Number	Case-Control Odds	Relative prevalence of sufficient-cause interaction	
				95% LCL^c^	LB^c^
Specific (*ADPRT* ^a^, *XRCC1* ^b^)					
(*Val/Val*, *Arg/Arg*)	157	186	0.8441	0.0000	0.0000
(*Val/Val*, *Arg/Gln*)	124	142	0.8732	0.0000	0.0000
(*Val/Val*, *Gln/Gln*)	26	31	0.8387	0.0000	0.0000
(*Val/Ala*, *Arg/Arg*)	273	286	0.9545	0.0000	0.0000
(*Val/Ala*, *Arg/Gln*)	180	183	0.9836	0.0000	0.0000
(*Val/Ala*, *Gln/Gln*)	56	53	1.0566	0.0000	0.0000
(*Ala/Ala*, *Arg/Arg*)	105	77	1.3636	0.0000	0.0000
(*Ala/Ala*, *Arg/Gln*)	59	55	1.0727	0.0000	0.0000
(*Ala/Ala*, *Gln/Gln*)	20	5	4.0000	0.0000	0.5221
Global	1000	1018	0.9823	0.0784	0.2471

^a^ *ADPRT*: *adenosine diphosphate ribosyltransferase*

^b^ *XRCC1*: *X-ray repair cross-complementing group 1*

^c^ *LCL* lower confidence limit for the lower bound, *LB* lower bound

## Discussion

Public health researchers have long sought a way to quantify sufficient-cause interactions using only the observational data at hand. Due to the non-identifiability problem, a sufficient-cause interaction can be tested but unfortunately not estimated. We are therefore provided with a very limited piece of information (of whether or not a sufficient-cause interaction is statistically significant), which falls far short of quantification. By setting bounds on sufficient-cause interactions (as demonstrated in the two examples in this paper), we can finally make some actual (if not exact) quantifications of such interactions.

Additional file 3 shows that the bounding formulae we presented in this paper produce ‘sharp’ bounds, i.e., bounds that are attainable. Previously, Sjölander et al. [20] derived an assumption-free lower bound for the cumulative completion risk of the specific class = *i*, *j* sufficient-cause interaction (which they called ‘weak’ sufficient-cause interaction). Using the notations of this paper, their bound is $$ \underset{\begin{array}{l}\left({i}^{\prime}\ne i\right)\in \left\{1,\dots, {L}_1\right\}\\ {}\left({j}^{\prime}\ne j\right)\in \left\{1,\dots, {L}_2\right\}\end{array}}{ \max}\left\{{\mathrm{Risk}}^{\mathrm{profile}= i, j}-{\mathrm{Risk}}^{\mathrm{profile}={i}^{\prime }, j}-{\mathrm{Risk}}^{\mathrm{profile}= i,{j}^{\prime }},0\right\}. $$ Additional file 4 shows we can achieve a sharper lower bound.

In this paper, the lower bound formulae also provide an avenue for testing of specific sufficient-cause interactions; if the bootstrapped 95% lower confidence limits for a particular lower bound is greater than zero, then the corresponding sufficient-cause interaction is present. Alternatively, one can rely on the lower bound for the global sufficient-cause interaction; if its bootstrapped 95% lower confidence limit is greater than zero, then some sufficient-cause interaction (between certain levels of the two factors) must be present. When both exposures are binary, such global test reduces to testing PRISM = 1 against PRISM ≠ 1 in cohort studies [7], and RERI = 0 against RERI ≠ 0 in case-control studies.

The assumption of no confounding is a strong one. To alleviate the problem, the data can be stratified by the confounders (if these are identified and measured in the study) and separate bounds set on sufficient-cause interactions using the proposed formulae in this paper for each of the resulting strata. Further work is warranted to develop stratified bounding methods for sufficient-cause interactions when the total number of strata is large and the average stratum size is small (the sparse-data scenario) and when some of the stratifying variables also interact with the two exposures of concern (sufficient-cause interactions involving more than two variables).

## Conclusions

The study provides bounding formulae for sufficient-cause interactions under the assumption of no redundancy. The bounds are sharp and sharper than previous bounds. Sufficient-cause interactions cannot be estimated but can be quantified using the bounds presented in this study.

## Additional files

Additional file 1: Derivations of the bounding formulas. (PDF 272 kb)

Additional file 2: R code. (PDF 151 kb)

Additional file 3: A proof that the bounds are sharp. (PDF 179 kb)

Additional file 4: A proof that the bounds are sharper than previous bounds. (PDF 286 kb)

## Abbreviations

*ADPRT*: *Adenosine diphosphate ribosyltransferas*e
BMI: Body mass index
LB: Lower bound
PRISM: Peril ratio index of synergy based on multiplicativity
RERI: Relative excess risk due to interaction
RP: Relative prevalence
UB: Upper bound
*XRCC1*: *X-ray repair cross-complementing group 1*
