# Supplementary material for: Sharp bounds on sufficient-cause interactions under the assumption of no redundancy
Source: BMC Med Res Methodol. 2017 Apr 21;17:71. doi: 10.1186/s12874-017-0348-y (PMC5399844; doi:10.1186/s12874-017-0348-y)
Supplement: Supplementary file 1 — Derivations of the bounding formulas. (PDF 272 kb) [file 12874_2017_348_MOESM1_ESM.pdf]

Additional file 1. Derivations of the bounding formulas.

Let $\text{Rate}^{\text{profile}=x_1,x_2}(t)$ denote the (instantaneous) disease rate at follow-up time t, for people in the population with $\text{profile}=x_1,x_2$. Let $\text{Rate}_{\text{class}=c_1,c_2}(t)$ denote the (instantaneous) arrival rate in the study population for the unknown components for the $\text{class}=c_1,c_2$ sufficient causes at follow-up time t (i.e., the completion rate for a $\text{class}=c_1,c_2$ at the time t). Under the no redundancy assumption, the disease rates and the completion rates are related through the following equations:

$$\text{Rate}^{\text{profile}=i,j}(t)=\text{Rate}_{\text{class}=*,*}(t)+\text{Rate}_{\text{class}=i,*}(t)+\text{Rate}_{\text{class}=*,j}(t)+\text{Rate}_{\text{class}=i,j}(t), \qquad \text{(A1.1)}$$

for $i\in\{1,...,L_1\}$ and $j\in\{1,...,L_2\}$.

Let $\text{Rate}_{\text{class}=\text{int}}(t)$ denote the rate of completion at a time t for any *interaction* class, and $\text{Rate}_{\text{class}=\text{any}}(t)$, the rate of completion at a time t for *any* class (all-unknown, main-effect, or interaction). Under the no redundancy assumption, we have that

$$\text{Rate}_{\text{class}=\text{int}}(t)=\sum_{i=1}^{L_1}\sum_{j=1}^{L_2}\text{Rate}_{\text{class}=i,j}(t) \qquad \text{(A1.2)}$$

and

$$\text{Rate}_{\text{class}=\text{any}}(t)=\text{Rate}_{\text{class}=*,*}(t)+\sum_{i=1}^{L_1}\text{Rate}_{\text{class}=i,*}(t)+\sum_{j=1}^{L_2}\text{Rate}_{\text{class}=*,j}(t)+\text{Rate}_{\text{class}=\text{int}}(t). \quad \text{(A1.3)}$$

The following derivations make frequent use of the peril index (PLoS ONE 2013;8: e67424). A ‘peril’ is identically a cumulative rate exponentiated, the reciprocal of a cumulative risk complement, and a one plus a cumulative odds, that is,

$$\text{Peril}=\exp\left[\int_0^T \text{Rate}(t)\times dt\right]=\left(1-\text{Risk}\right)^{-1}=1+\text{Odds}, \qquad \text{(A1.4)}$$

and is an index no less than 1.

The cumulative completion risk over (0, T) for a specific $\text{class}=i,j$ sufficient-cause interaction is

$$\text{Risk}_{\text{class}=i,j}=1-\text{Peril}_{\text{class}=i,j}^{-1}=1-\exp\left[-\int_0^T \text{Rate}_{\text{class}=i,j}(t)dt\right], \tag{A1.5}$$

for $i\in\{1,...,L_1\}$ and $j\in\{1,...,L_2\}$. The cumulative completion risk over (0, T) for the global sufficient-cause interaction is

$$\text{Risk}_{\text{class}=\text{int}}=1-\text{Peril}_{\text{class}=\text{int}}^{-1}=1-\exp\left[-\int_0^T \text{Rate}_{\text{class}=\text{int}}(t)dt\right]. \tag{A1.6}$$

The cumulative completion risk over (0, T) for any class is

$$\text{Risk}_{\text{class}=\text{any}}=1-\text{Peril}_{\text{class}=\text{any}}^{-1}=1-\exp\left[-\int_0^T \text{Rate}_{\text{class}=\text{any}}(t)dt\right]. \tag{A1.7}$$

To set a lower bound on $\text{Risk}_{\text{class}=i,j}$ ($i\in\{1,...,L_1\}$, $j\in\{1,...,L_2\}$), we first establish the following inequalities from (A1.1):

$$\begin{aligned}\text{Peril}_{\text{class}=i,j}&=\exp\left[\int_0^T \text{Rate}_{\text{class}=i,j}(t)dt\right]\\&\geq\exp\left[\int_0^T\left[\text{Rate}^{\text{profile}=i,j}(t)-\text{Rate}^{\text{profile}=i',j}(t)-\text{Rate}^{\text{profile}=i,j'}(t)\right]dt\right]\\&=\frac{\exp\left[\int_0^T \text{Rate}^{\text{profile}=i,j}(t)dt\right]}{\exp\left[\int_0^T \text{Rate}^{\text{profile}=i',j}(t)dt\right]\times\exp\left[\int_0^T \text{Rate}^{\text{profile}=i,j'}(t)dt\right]}\\&=\frac{\text{Peril}^{\text{profile}=i,j}}{\text{Peril}^{\text{profile}=i',j}\times\text{Peril}^{\text{profile}=i,j'}},\end{aligned} \tag{A1.8}$$

for any $(i'\neq i)\in\{1,...,L_1\}$ and $(j'\neq j)\in\{1,...,L_2\}$. Therefore

$$\begin{aligned}\text{Risk}_{\text{class}=i,j}^{\text{LB}}&=1-\max_{\substack{(i'\neq i)\in\{1,...,L_1\}\\(j'\neq j)\in\{1,...,L_2\}}}{}^{-1}\left\{\frac{\text{Peril}^{\text{profile}=i,j}}{\text{Peril}^{\text{profile}=i',j}\times\text{Peril}^{\text{profile}=i,j'}},\ 1\right\}\\&=1-\min_{\substack{(i'\neq i)\in\{1,...,L_1\}\\(j'\neq j)\in\{1,...,L_2\}}}\left\{\frac{1-\text{Risk}^{\text{profile}=i,j}}{\left(1-\text{Risk}^{\text{profile}=i',j}\right)\times\left(1-\text{Risk}^{\text{profile}=i,j'}\right)},\ 1\right\}.\end{aligned} \tag{A1.9}$$

To set an upper bound on $\text{Risk}_{\text{class}=i,j}$ ($i\in\{1,...,L_1\}$, $j\in\{1,...,L_2\}$), Again from (A1.1), we have

$$\text{Rate}_{\text{class}=i,j}(t)\leq\text{Rate}^{\text{profile}=i,j}(t) \tag{A1.10}$$

for all t in (0, T), which implies $\text{Peril}_{\text{class}=i,j} \le \text{Peril}^{\text{profile}=i,j}$. Therefore

$$\text{Risk}^{\text{UB}}_{\text{class}=i,j} = \text{Risk}^{\text{profile}=i,j}. \qquad \text{(A1.11)}$$

To set a lower bound on $\text{Risk}_{\text{class=int}}$, we first establish the following inequalities with the use of contrast coefficients described in the text. Again from (A1.1), we have

$$\begin{aligned}\text{Peril}_{\text{class=int}} &= \exp\left\{\int_0^T \left[\sum_{i=1}^{L_1}\sum_{j=1}^{L_2} \text{Rate}_{\text{class}=i,j}(t)\right]dt\right\} \\ &\ge \exp\left\{\int_0^T \left[\sum_{i=1}^{L_1}\sum_{j=1}^{L_2} u_i \times v_j \times \text{Rate}_{\text{class}=i,j}(t)\right]dt\right\} \\ &= \exp\left\{\int_0^T \left[\sum_{i=1}^{L_1}\sum_{j=1}^{L_2} u_i \times v_j \times \text{Rate}^{\text{profile}=i,j}(t)\right]dt\right\} \\ &= \prod_{i=1}^{L_1}\prod_{j=1}^{L_2}\left(\text{Peril}^{\text{profile}=i,j}\right)^{u_i \times v_j},\end{aligned} \qquad \text{(A1.12)}$$

for any legitimate contrast coefficients $(u_1,...,u_{L_1})$ and $(v_1,...,v_{L_2})$. Therefore,

$$\begin{aligned}\text{Risk}^{\text{LB}}_{\text{class=int}} &= 1 - \max_{\substack{\text{permutations of } (u_1,...,u_{L_1}) \\ \text{and permutations of } (v_1,...,v_{L_2})}}^{-1} \left\{\prod_{i=1}^{L_1}\prod_{j=1}^{L_2}\left(\text{Peril}^{\text{profile}=i,j}\right)^{u_i \times v_j},\ 1\right\} \\ &= 1 - \min_{\substack{\text{permutations of } (u_1,...,u_{L_1}) \\ \text{and permutations of } (v_1,...,v_{L_2})}} \left\{\prod_{i=1}^{L_1}\prod_{j=1}^{L_2}\left(1-\text{Risk}^{\text{profile}=i,j}\right)^{u_i \times v_j},\ 1\right\}.\end{aligned} \qquad \text{(A1.13)}$$

To set an upper bound on $\text{Risk}_{\text{class=int}}$, from (A1.1)~(A1.3) we have

$$\text{Rate}_{\text{class=int}}(t) \le \text{Rate}_{\text{class=any}}(t) \le \sum_{i=1}^{L_1}\sum_{j=1}^{L_2} \text{Rate}^{\text{profile}=i,j}(t) \qquad \text{(A1.14)}$$

for all t in (0, T), which implies $\text{Peril}_{\text{class=int}} \le \text{Peril}_{\text{class=any}} \le \prod_{i=1}^{L_1}\prod_{j=1}^{L_2}\text{Peril}^{\text{profile}=i,j}$. Therefore

$$\text{Risk}^{\text{UB}}_{\text{class=int}} = 1 - \left(\prod_{i=1}^{L_1}\prod_{j=1}^{L_2}\text{Peril}^{\text{profile}=i,j}\right)^{-1} = 1 - \prod_{i=1}^{L_1}\prod_{j=1}^{L_2}\left(1-\text{Risk}^{\text{profile}=i,j}\right). \qquad \text{(A1.15)}$$

Finally, the bounds on the relative prevalence of sufficient-cause interactions are functions of the above bounds on the cumulative completion risks, as presented in the text.
