# Supplementary material for: Sharp bounds on sufficient-cause interactions under the assumption of no redundancy
Source: BMC Med Res Methodol. 2017 Apr 21;17:71. doi: 10.1186/s12874-017-0348-y (PMC5399844; doi:10.1186/s12874-017-0348-y)
Supplement: Supplementary file 2 — R code. (PDF 151 kb) [file 12874_2017_348_MOESM2_ESM.pdf]

Additional file 2. R code.

```
############################################################
#                                                          #
# Arguments for the function, bounds.cohort:               #
#   case: the numbers of cases, a matrix                   #
#   population: the numbers of populations, a matrix       #
#   bootstrap: the total number of bootstrapping, a scalar #
#              (default=10000)                             #
#                                                          #
#                                                          #
# Arguments for the function, bounds.cscn:                 #
#   case: the numbers of cases, a matrix                   #
#   control: the numbers of controls, a matrix             #
#   bootstrap: the total number of bootstrapping, a scalar #
#              (default=10000)                             #
#                                                          #
############################################################
bounds.cohort=function(case, population, bootstrap=10000){
 L1=nrow(case)
 half=floor(L1/2)
 double=half*2
 small=2^half -1
 large=2^double - 2^half
 u={}
 for (i in small:large){
   bits=as.integer( intToBits(i)[1:double])
   if (sum(bits)==half) u=rbind(u,2*bits-1)
 }
 if (double != L1){
   plate=u
   u=cbind(0,plate)
   for (i in 1:(double-1)){
     u=rbind(u,cbind(plate[,1:i],0,plate[,(i+1):double]))
   }
   u=rbind(u,cbind(plate,0))
 }
 perm.u=nrow(u)
```

```
L2=ncol(case)
half=floor(L2/2)
double=half*2
small=2^half -1
large=2^double - 2^half
v={}
for (i in small:large){
  bits=as.integer( intToBits(i)[1:double])
  if (sum(bits)==half) v=cbind(v,2*bits-1)
}
if (double != L2){
  plate=v
  v=rbind(0,plate)
  for (i in 1:(double-1)){
     v=cbind(v,rbind(plate[1:i,],0,plate[(i+1):double,]))
  }
  v=cbind(v,rbind(plate,0))
}
perm.v=ncol(v)
sum.case=sum(case)
sum.population=sum(population)
ave.risk=sum.case/sum.population
risk=case/population
risk1=1-risk
zero=1e-50
infinity=1e100
perm=matrix(1, nrow=perm.u, ncol=perm.v)
for (i2 in 1:perm.u){
for (j2 in 1:perm.v){
   positive=1
   negative=1
   for (i in 1:L1){
   for (j in 1:L2){
      temp=u[i2,i]*v[j,j2]
      if (temp==1)  positive=positive*risk1[i,j]
      if (temp==-1) negative=negative*risk1[i,j]
   }}
   perm[i2,j2]=(positive+zero)/(negative+zero)
```

```
}}
mini=min(perm)
posi=which(perm==mini, arr.ind=TRUE)
minu=posi[1,1]
minv=posi[1,2]
u=u[minu,]
v=v[,minv]
Risk.int.LB=1-min(mini,1)
Risk.int.UB=1-prod(risk1)
RP.int.LB=ifelse(Risk.int.UB==0, 0, Risk.int.LB/Risk.int.UB)
Risk.ij.LB=matrix(nrow=L1, ncol=L2)
rowmin=matrix(nrow=L1, ncol=L2)
colmin=matrix(nrow=L1, ncol=L2)
for (i in 1:L1){
for (j in 1:L2){
  temp=matrix(infinity, nrow=L1, ncol=L2)
  for (i2 in 1:L1){
  for (j2 in 1:L2){
    if ((i2!=i)&(j2!=j)){
      negative=risk1[i2,j]*risk1[i,j2]
      temp[i2,j2]=(risk1[i,j]+zero)/(negative+zero)
    }
  }}
  mini=min(temp)
  posi=which(temp==mini, arr.ind=TRUE)
  rowmin[i,j]=posi[1,1]
  colmin[i,j]=posi[1,2]
  Risk.ij.LB[i,j]=1-min(mini,1)
}}
RP.ij.LB=ifelse(risk==0, 0, Risk.ij.LB/risk)
case.b=matrix(nrow=L1, ncol=L2)
one=matrix(1,nrow=L1,ncol=L2)
Risk.int.LB.b=1:bootstrap
Risk.int.UB.b=1:bootstrap
L12=L1*L2
Risk.ij.LB.b=matrix(nrow=L12, ncol=bootstrap)
Risk.ij.UB.b=matrix(nrow=L12, ncol=bootstrap)
RP.ij.LB.b=matrix(nrow=L12, ncol=bootstrap)
```

```
for (simu in 1:bootstrap){
  case.b=rbinom(prob=risk, n=one, size=population)
  risk.b=case.b/population
  risk1.b=1-risk.b
  positive=1
  negative=1
  for (i in 1:L1){
  for (j in 1:L2){
     index=L2*(i-1)+j
     temp=u[i]*v[j]
     if (temp==1)  positive=positive*risk1.b[i,j]
     if (temp==-1) negative=negative*risk1.b[i,j]
     neg_ij=risk1.b[rowmin[i,j],j]*risk1.b[i,colmin[i,j]]
     temp=(risk1.b[i,j]+zero)/(neg_ij+zero)
     Risk.ij.LB.b[index,simu]=1-min(temp,1)
     Risk.ij.UB.b[index,simu]=risk.b[i,j]
  }}
  temp=(positive+zero)/(negative+zero)
  Risk.int.LB.b[simu]=1-min(temp,1)
  Risk.int.UB.b[simu]=1-prod(risk1.b)
}
RP.int.LB.b=
   ifelse(Risk.int.UB.b==0, 0, Risk.int.LB.b / Risk.int.UB.b)
RP.ij.LB.b=
   ifelse(Risk.ij.UB.b==0, 0, Risk.ij.LB.b / Risk.ij.UB.b)
Risk.global=matrix(nrow=1, ncol=7)
colnames(Risk.global)=c(" CS", " POP", " RISK",
                        " 95%LCL", " LB",
                        " UB", " 95%UCL")
Risk.global[1,1]=sum.case
Risk.global[1,2]=sum.population
Risk.global[1,3]=ave.risk
Risk.global[1,4]=quantile(Risk.int.LB.b, prob=0.025)
Risk.global[1,5]=Risk.int.LB
Risk.global[1,6]=Risk.int.UB
Risk.global[1,7]=quantile(Risk.int.UB.b, prob=0.975)
RP.global=matrix(nrow=1, ncol=5)
colnames(RP.global)=c(" CS", " POP", " RISK",
```

```
                     "95%LCL", " LB")
RP.global[1,1]=sum.case
RP.global[1,2]=sum.population
RP.global[1,3]=ave.risk
RP.global[1,4]=quantile(RP.int.LB.b, prob=0.05)
RP.global[1,5]=RP.int.LB
 Risk.specific=matrix(nrow=L12, ncol=9)
colnames(Risk.specific)=c(" X1", " X2",
                          " CS", " POP", " RISK",
                          " 95%LCL", " LB",
                          " UB", " 95%UCL")
RP.specific=matrix(nrow=L12, ncol=7)
colnames(RP.specific)=c(" X1", " X2",
                        " CS", " POP", " RISK",
                        " 95%LCL", " LB")
for (i in 1:L1){
for (j in 1:L2){
  index=L2*(i-1)+j
  Risk.specific[index,1]=i
  Risk.specific[index,2]=j
  Risk.specific[index,3]=case[i,j]
  Risk.specific[index,4]=population[i,j]
  Risk.specific[index,5]=risk[i,j]
  Risk.specific[index,6]=quantile(Risk.ij.LB.b[index,],
                                  prob=0.025)
  Risk.specific[index,7]=Risk.ij.LB[i,j]
  Risk.specific[index,8]=risk[i,j]
  Risk.specific[index,9]=quantile(Risk.ij.UB.b[index,],
                                  prob=0.975)
  RP.specific[index,1]=i
  RP.specific[index,2]=j
  RP.specific[index,3]=case[i,j]
  RP.specific[index,4]=population[i,j]
  RP.specific[index,5]=risk[i,j]
  RP.specific[index,6]=quantile(RP.ij.LB.b[index,],
                                  prob=0.05)
  RP.specific[index,7]=RP.ij.LB[i,j]
}}
```

```
 RESULTS=list(Risk.global=Risk.global,
              RP.global=RP.global,
              Risk.specific=Risk.specific,
              RP.specific=RP.specific)
 return(RESULTS)
}

bounds.cscn=function(case, control, bootstrap=10000){
 L1=nrow(case)
 half=floor(L1/2)
 double=half*2
 small=2^half -1
 large=2^double - 2^half
 u={}
 for (i in small:large){
   bits=as.integer( intToBits(i)[1:double])
   if (sum(bits)==half) u=rbind(u,2*bits-1)
 }
 if (double != L1){
   plate=u
   u=cbind(0,plate)
   for (i in 1:(double-1)){
      u=rbind(u,cbind(plate[,1:i],0,plate[,(i+1):double]))
   }
   u=rbind(u,cbind(plate,0))
 }
 perm.u=nrow(u)
 L2=ncol(case)
 half=floor(L2/2)
 double=half*2
 small=2^half -1
 large=2^double - 2^half
 v={}
 for (i in small:large){
   bits=as.integer( intToBits(i)[1:double])
   if (sum(bits)==half) v=cbind(v,2*bits-1)
 }
 if (double != L2){
```

```
  plate=v
  v=rbind(0,plate)
  for (i in 1:(double-1)){
    v=cbind(v,rbind(plate[1:i,],0,plate[(i+1):double,]))
  }
  v=cbind(v,rbind(plate,0))
}
perm.v=ncol(v)
infinity=1e50
impossible=-1e100
sum.case=sum(case)
sum.control=sum(control)
pop=case+control
risk=case/pop
odds=ifelse(control==0, infinity, case/control)
perm=matrix(1, nrow=perm.u, ncol=perm.v)
for (i2 in 1:perm.u){
for (j2 in 1:perm.v){
   positive=0
   negative=0
   for (i in 1:L1){
   for (j in 1:L2){
      temp=u[i2,i]*v[j,j2]
      if (temp==1)  positive=positive+odds[i,j]
      if (temp==-1) negative=negative+odds[i,j]
   }}
   perm[i2,j2]=ifelse(positive>infinity, infinity, positive)-
               ifelse(negative>infinity, infinity, negative)
}}
maxi=max(perm)
posi=which(perm==maxi, arr.ind=TRUE)
maxu=posi[1,1]
maxv=posi[1,2]
u=u[maxu,]
v=v[,maxv]
all=sum(odds)
RP.int.LB=max(maxi,0) / ifelse(all>infinity, infinity, all)
RP.ij.LB=matrix(nrow=L1, ncol=L2)
```

```
rowmax=matrix(nrow=L1, ncol=L2)
colmax=matrix(nrow=L1, ncol=L2)
for (i in 1:L1){
for (j in 1:L2){
   temp=matrix(impossible, nrow=L1, ncol=L2)
   for (i2 in 1:L1){
   for (j2 in 1:L2){
     if ((i2!=i)&(j2!=j)){
        negative=odds[i2,j]+odds[i,j2]
        temp[i2,j2]=
          ifelse(odds[i,j]>infinity, infinity, odds[i,j])-
          ifelse(negative>infinity, infinity, negative)
     }
   }}
   maxi=max(temp)
   posi=which(temp==maxi, arr.ind=TRUE)
   rowmax[i,j]=posi[1,1]
   colmax[i,j]=posi[1,2]
   RP.ij.LB[i,j]=ifelse(odds[i,j]==0,0,max(maxi,0)/odds[i,j])
}}
case.b=matrix(nrow=L1, ncol=L2)
one=matrix(1,nrow=L1,ncol=L2)
RP.int.LB.b=1:bootstrap
L12=L1*L2
RP.ij.LB.b=matrix(nrow=L12, ncol=bootstrap)
for (simu in 1:bootstrap){
  case.b=rbinom(prob=risk, n=one, size=pop)
  control.b=pop-case.b
  odds.b=ifelse(control.b==0, infinity, case.b/control.b)
  positive=0
  negative=0
  for (i in 1:L1){
  for (j in 1:L2){
      index=L2*(i-1)+j
      temp=u[i]*v[j]
      if (temp==1)  positive=positive+odds.b[i,j]
      if (temp==-1) negative=negative+odds.b[i,j]
      neg_ij=odds.b[rowmax[i,j],j] + odds.b[i,colmax[i,j]]
```

```
      diff=ifelse(odds.b[i,j]>infinity, infinity, odds.b[i,j])-
           ifelse(neg_ij>infinity, infinity, neg_ij)
      RP.ij.LB.b[index,simu]=
              ifelse(odds.b[i,j]==0, 0, max(diff,0)/odds.b[i,j])
  }}
  diff=ifelse(positive>infinity, infinity, positive)-
          ifelse(negative>infinity, infinity, negative)
  all=sum(odds.b)
  RP.int.LB.b[simu]=max(diff,0) /
                        ifelse(all>infinity, infinity, all)
 }
 RP.global=matrix(nrow=1, ncol=5)
 colnames(RP.global)=c(" CS", " CN", " ODDS",
                        " 95%LCL", " LB")
 RP.global[1,1]=sum.case
 RP.global[1,2]=sum.control
 RP.global[1,3]=sum.case/sum.control
 RP.global[1,4]=quantile(RP.int.LB.b, prob=0.05)
 RP.global[1,5]=RP.int.LB
 RP.specific=matrix(nrow=L12, ncol=7)
 colnames(RP.specific)=c(" X1", " X2",
                           " CS", " CN", " ODDS",
                           " 95%LCL", " LB")
 for (i in 1:L1){
 for (j in 1:L2){
   index=L2*(i-1)+j
   RP.specific[index,1]=i
   RP.specific[index,2]=j
   RP.specific[index,3]=case[i,j]
   RP.specific[index,4]=control[i,j]
   RP.specific[index,5]=odds[i,j]
   RP.specific[index,6]=quantile(RP.ij.LB.b[index,],
                                        prob=0.05)
   RP.specific[index,7]=RP.ij.LB[i,j]
 }}
 RESULTS=list(RP.global=RP.global, RP.specific=RP.specific)
 return(RESULTS)
}
```

```
############################################################
# Example 1. A Cohort Study on Hypertension Risk          #
############################################################

example1.diseased=
  t(matrix( c(79,100,153,278), nrow=2))
example1.nondiseased=
  t(matrix( c(1731,581,1232,743), nrow=2))
example1.population=example1.diseased + example1.nondiseased
bounds.cohort( case= example1.diseased,
               population= example1.population )

############################################################
# Example 2. A Case-Control Study on Lung Cancer Risk     #
############################################################
example2.case=
  t(matrix( c(157,124,26,273,180,56,105,59,20), nrow=3))
example2.control=
  t(matrix(c(186,142,31,286,183,53,77,55,5), nrow=3))
bounds.cscn( case= example2.case,
             control= example2.control )
```
