# Supplementary material for: Sharp bounds on sufficient-cause interactions under the assumption of no redundancy
Source: BMC Med Res Methodol. 2017 Apr 21;17:71. doi: 10.1186/s12874-017-0348-y (PMC5399844; doi:10.1186/s12874-017-0348-y)
Supplement: Supplementary file 3 — A proof that the bounds are sharp. (PDF 179 kb) [file 12874_2017_348_MOESM3_ESM.pdf]

Additional file 3. A proof that the bounds are sharp.

To prove that the bounds on sufficient-cause interactions as presented in text are indeed sharp bounds, it suffices to show that all the inequalities in Web Appendix 1 are attainable:

A1.8 can be attained when

$$\text{Rate}_{\text{class}=i',j}(t)=\text{Rate}_{\text{class}=i,j'}(t)=\text{Rate}_{\text{class}=i',*}(t)=\text{Rate}_{\text{class}=*,j'}(t)=\text{Rate}_{\text{class}=*,*}(t)=0$$

for $(i'\neq i)\in\{1,...,L_1\}$ and $(j'\neq j)\in\{1,...,L_2\}$;

A1.10 and A1.14 can be attained when

$$\text{Rate}_{\text{class}=i,*}(t)=\text{Rate}_{\text{class}=*,j}(t)=\text{Rate}_{\text{class}=*,*}(t)=0 \quad \text{for } i\in\{1,...,L_1\} \text{ and } j\in\{1,...,L_2\};$$

A1.12 can be attained when

$$\text{Rate}_{\text{class}=i,j}(t)=0 \quad \text{if } u_i\times v_j\neq 1.$$
