# Supplementary material for: Sharp bounds on sufficient-cause interactions under the assumption of no redundancy
Source: BMC Med Res Methodol. 2017 Apr 21;17:71. doi: 10.1186/s12874-017-0348-y (PMC5399844; doi:10.1186/s12874-017-0348-y)
Supplement: Supplementary file 4 — A proof that the bounds are sharper than previous bounds. (PDF 286 kb) [file 12874_2017_348_MOESM4_ESM.pdf]

Additional file 4. A proof that the bounds are sharper than previous bounds.

For the lower bounds:

$$\begin{aligned}
\text{Risk}^{\text{LB}}_{\text{class}=i,j} &= 1-\min_{\substack{(i'\neq i)\in\{1,\ldots,L_1\}\\(j'\neq j)\in\{1,\ldots,L_2\}}}\left\{\frac{1-\text{Risk}^{\text{profile}=i,j}}{\left(1-\text{Risk}^{\text{profile}=i',j}\right)\times\left(1-\text{Risk}^{\text{profile}=i,j'}\right)},\ 1\right\}\\
&= \max_{\substack{(i'\neq i)\in\{1,\ldots,L_1\}\\(j'\neq j)\in\{1,\ldots,L_2\}}}\left\{\frac{\text{Risk}^{\text{profile}=i,j}-\text{Risk}^{\text{profile}=i',j}-\text{Risk}^{\text{profile}=i,j'}+\text{Risk}^{\text{profile}=i',j}\times\text{Risk}^{\text{profile}=i,j'}}{\left(1-\text{Risk}^{\text{profile}=i',j}\right)\times\left(1-\text{Risk}^{\text{profile}=i,j'}\right)},\ 0\right\}\\
&\geq \max_{\substack{(i'\neq i)\in\{1,\ldots,L_1\}\\(j'\neq j)\in\{1,\ldots,L_2\}}}\left\{\text{Risk}^{\text{profile}=i,j}-\text{Risk}^{\text{profile}=i',j}-\text{Risk}^{\text{profile}=i,j'},\ 0\right\}\\
&= \text{Sjölander et al.'s lower bound.}
\end{aligned}$$

For the upper bounds:

Sjölander et al. (21) also derived an assumption-free upper bound for the specific interaction:

$\min\left\{\text{Risk}^{\text{profile}=i,j},\ \sum_{(i'\neq i)\in\{1,\ldots,L_1\}}\left(1-\text{Risk}^{\text{profile}=i',j}\right),\ \sum_{(j'\neq j)\in\{1,\ldots,L_2\}}\left(1-\text{Risk}^{\text{profile}=i,j'}\right)\right\}$. However, this bound is wrongly too sharp. In fact, the cumulative completion risk of the $\text{class}=i,j$ sufficient-cause interaction is bounded above, neither by $\sum_{(i'\neq i)\in\{1,\ldots,L_1\}}\left(1-\text{Risk}^{\text{profile}=i',j}\right)$ nor by $\sum_{(j'\neq j)\in\{1,\ldots,L_2\}}\left(1-\text{Risk}^{\text{profile}=i,j'}\right)$, as dictated by Sjölander et al (21). To see why, let us assume that all the diseased subjects in a population are caused by and only by sufficient-cause interactions, that is, all the $\text{profile}=i,j$ diseased subjects had contracted the disease because of the completion of the $\text{class}=i,j$ sufficient-cause interaction, for all $i\in\{1,\ldots,L_1\}$ and all $j\in\{1,\ldots,L_2\}$. Further, assume that the follow-up is very long (T is very large) so that $\text{Risk}^{\text{profile}=i,j}\approx 1$ for all $i\in\{1,\ldots,L_1\}$ and all $j\in\{1,\ldots,L_2\}$. Sjölander et al.'s upper bound (21) then becomes nearly zero. Clearly, it should not be.
